# Should we stay or should we go? Recent insights on drug discontinuation in multiple sclerosis

**DOI:** 10.1186/s42466-025-00379-y

**Published:** 2025-04-21

**Authors:** Anne Mrochen, Sven G. Meuth, Steffen Pfeuffer

**Affiliations:** 1 Department of Neurology, University Hospital Giessen and Marburg, Justus-Liebig-University Giessen, Klinikstr. 33, 35392 Giessen, Germany; 2 Department of Neurology, University Hospital Duesseldorf, Heinrich-Heine-University, Duesseldorf, Germany

**Keywords:** Multiple sclerosis, Treatment discontinuation, Cardiovascular disease, Ageing

## Abstract

**Background:**

The decision to discontinue disease-modifying therapies (DMTs) in patients with multiple sclerosis (PwMS) is a critical clinical challenge. Historically, DMTs were discontinued due to side effects, treatment limitations, or progression to secondary progressive MS. However, advancements in MS therapies, particularly high-efficacy DMTs (HE-DMTs) and the increased knowledge on disease courses and phenotypes have resulted in more personalized treatment approaches and introduced discussion on scheduled DMT discontinuation. This review explores the current evidence on DMT discontinuation, focusing on its implications for aging populations and the interplay between cardiovascular diseases (CVD) and MS.

**Current evidence and interplay with CVD:**

Randomized trials such as DISCOMS and DOT-MS have provided insights into discontinuing DMTs in stable patients. In summary, both randomized clinical trials highlight the risk of disease reactivation following treatment discontinuation. Due to the limited sample size, neither study was able to conduct subgroup analyses based on age groups. Additionally, DOT-MS was terminated prematurely, direct comparisons with other studies should be avoided. While older studies and observational data (e.g., OFSEP) have shown relapse risks associated with discontinuation, particularly for drugs like natalizumab and fingolimod, there is limited data on HE-DMT discontinuation outcomes. Comorbidities, particularly CVDs, further complicate decisions regarding the continuation of DMTs in older adults. MS patients bear a higher burden of CVD, which is also associated with unfavorable disease courses. While optimizing cardiovascular risk profiles appears advisable, it remains unclear whether DMTs themselves have a positive impact on CVDs.

**Conclusion:**

Given the complexities associated with discontinuing DMTs in MS patients, it is essential to balance the avoidance of polypharmacy with the potential risks of disease reactivation and the impact of comorbidities, especially CVDs, on disease progression. The interplay between MS and CVD highlights the importance of a holistic risk assessment when considering DMT discontinuation.

## Background

The decision to discontinue DMTs in PwMS has become a critical clinical issue that has previously been discussed in the literature [1, 2].

In the past, DMT were stopped for various reasons including side effects (especially glatiramer acetate or beta-interferons because of their injection site reactions), dosing limitations (exposure limits for mitoxantrone; treatment duration for natalizumab) or development of a secondary progressive disease course [3–5].

However, the treatment landscape has dramatically changed throughout the last year now including various substances thus allowing a more personalized therapeutic approach for PwMS. Several new HE-DMT such as the CD20-antibodies, S1P receptor modulators were introduced and the safety profile of natalizumab has been developed further thus allowing long-term treatment in a relevant proportion of patients [6].

These new DMT have been broadly incorporated in clinical routine and several studies have shown that their early use results in favorable long-term outcomes [7, 8]. Additionally, the previously observed seven years shorter live expectancy in PwMS than the general population, is expected to normalize [9–11]. This trend, combined with a relatively stable incidence rate, is anticipated to contribute to an increase in the prevalence of MS as patients experience longer lifespans [12]. Consequently, an increasing number of PwMS, including older individuals, are receiving highly effective therapies, and this trend is expected to persist.

The evolution of MS with age is well accepted and it is logical to hypothesize that the effectiveness of DMTs may vary as individual’s age and the pathogenesis of the disease changes [11, 13]. As the benefits of currently available disease-modifying therapies diminish with advancing age, longer disease duration, and the transition from a relapsing to a more progressive/neurodegenerative phenotype, the side effects and associated risks of these treatments may become more significant [14]. Older adults, particularly those with substantial disabilities, experience increased rates of infections [15]. PwMS on DMTs face an elevated risk of infections compared to the general population, and this risk is partly dependent on the choice of treatment [16].

These effects have to be well-weighted in the context of immunosenescence, which itself may hamper the immune function. For example, the incidence of herpes zoster manifestations profoundly increases in PwMS older than 50 years and this was observed in patients with lymphopenia following cladribine treatment as well [17, 18]. Moreover, increased side effects in ocrelizumab-treated patients from the long-term follow-up of the ORATORIO trial (primary progressive MS, mean age at baseline: 44.4 ± 8.3 years) compared to the OPERA trials (relapsing MS, mean age at baseline: 37.2 ± 9.2 years) further underline age-related risks of DMT in PwMS [19, 20]. Within recently presented 11-year follow-up data, 6.7 serious adverse events (per 100 patient-years) were observed in ocrelizumab-exposed OPERA patients compared to 12.2 events in ORATORIO in this included 2.5 serious infections in OPERA compared to 5,1 in ORATORIO [21].

Ideally, randomized controlled trials would encompass all age groups and multiple sclerosis phenotypes to support evidence-based decision-making. However, many phase-three clinical trials typically impose an upper age limit of 55 years, resulting in a substantial gap in safety and efficacy data for older patient groups, despite the fact that over 35% of adults with MS are above 55 years of age [22, 23].

Consequently, it appears inevitable to develop protocols for DMT cessation or therapy sequencing in PwMS and identification of the circumstances for a “safe stop” appears as one of the pivotal tasks in clinical MS research throughout the next years. As we have already introduced comorbidities and side effects as prominent reasons for discontinuation of DMT, we will also highlight the potentially reciprocal association of these comorbidities and MS progression. Already, some studies were conducted within past years in order to further elucidate whether the cessation of DMT is safe in PwMS [24, 25]. We will briefly introduce these studies and will discuss their implications and limitations within this review article.

## Current evidence: discontinuation of treatment

As stated above, the wish for stopping DMTs among patients accompanies neurologists nearly as long as DMTs are available. Consequently, almost all national and international guidelines incorporated statements on this procedure yet almost entirely deemed this “individual decision” based upon informed consent although attempts to develop risk stratification scores were made [26, 27]. Notably, among qualified 22 publications, 12 publications presented actual studies whereas 10 publications provided practice recommendations [28, 29]. However, most of the “previous studies” focused on specific situations such as the “rebound” phenomenon in patients stopping natalizumab or fingolimod. However, these studies did not evaluate long-term outcomes in absence of DMT but aimed on development of short-term mitigation strategies [30, 31] or development of useful treatment sequences [32]. Additionally, cessation of these drugs was not driven by patients’ wishes but safety concerns (e.g., increased JCV titres) and thus was often made independently from disease activity. Additionally, previous studies were limited by their heterogeneous patient collectives including both relapsing and non-relapsing forms of MS [28]. Furthermore, different studies define "stability" in MS variably, with some considering MRI activity and others focusing on relapse history. This variability in definitions may impact the generalizability of study findings. Finally, most of these studies were compromised by their potential selection bias. Although some studies included propensity-score matching in an attempt to improve comparability [33], it ignores unknown confounders that likely contributed to treatment decisions.

Thus, two randomized controlled clinical trials were initiated in 2020 (DOT-MS; NCT04260711) and 2021 (DISCOMS; NCT04754542), Table 1. The primary objective of both studies was to evaluate whether discontinuation of first-line DMT was non-inferior to continuation in patients with stable relapsing MS. Patients were deemed “stable” and thus eligible if they had no relapses within the past five years and no new MRI lesions (DISCOMS: five years; DOT-MS: three years). Presence of disability worsening within these periods was not considered upon enrolment and both studies were open to relapsing–remitting MS as well as to secondary-progressive MS as long as patients were on treatment. DISCOMS was restricted to patients older than 55 year of age whereas DOT-MS included patients older than 18 years of age already [24, 25]. Patients were randomized 1:1 to either continue or stop their treatment. DISCOMS was designed for a 24-month study period whereas DOT-MS was scheduled over 42 months.

**Table 1:** Summary of key findings, study populations, and sample sizes from the DOT-MS and DISCOMS RCTs and the OFSEP study

Design	DISCOMS		DOT-MS		OFSEP study	
	Corboy et al. [25]		Coerver et al. [24]		Jouvenot et al. [34]	
	RCT		RCT		Registry study; 1:1 PS-Matched	
	Continuation	Discontinuation	Continuation	Discontinuation	Continuation	Discontinuation
Patients, number	128	131	44	45	154	154
Age, years^#^	62 (59–68)	63 (59–67)	55.0 (50.0–59.0)	54.0 (47.0–58.0)	57.9 (5.2)	57.5 (5.9)
Years since MS onset^#^	20.9 (10.4)	23.4 (11)	13.3 (9.9–22.2)	14.1 (9.4–19.6)	19.2 (14.6–25.8)	20.5 (14.7–26.1)
Baseline EDSS^#^	3.3 (1.8)	3.4 (1.8)	3.1 (1.6)	3.1 (2.0)	5 (3.5–6.0)	4.5 (3.5–6.0)
Years since last relapse^#^	13.2 (6.2)	14.5 (7)	9.8 (6.8–13.3)	9.4 (7.1–12.3)	4.7 (3.3–6.4)	4.4 (3.1–6.6)
Follow up time, months^#^	24.5 (21.4–25.2)		15.3 (11.4–23.9)		23 (23)	36 (26)
Primary Endpoint	Relapse or 1 T2 brain MRI lesion over 2 years		Relapse and/or ≥ 3 T2 brain MRI lesion or ≥ 2 contrast-enhancing lesions		Registry Study; Outcomes: Time to relapse, MRI-activity or to confirmed disability progression (CDP)	
Primary outcome event (no./total no. (%))	6/128 (4.7%)	16/131 (12.2%)	0/44 (0%)	8/45 (17.8%)	Relapse (HR, 95% CI): 4.1, 2.0–8.5 MRI-activity (HR, 95% CI): 3.6, 2.0–6.5 CDP (HR, 95% CI): 2.6; 1.5–4.4	

^#^Mean ± SD or median (interquartile range: 25th–75th percentile)

DISCOMS randomized 259 patients (128 continuers; 131 stoppers) with a median age of 62/63 years, a median disease duration of 20.9/23.4 years and a median since the last relapse of 13.2/14.5 years. Of note, only 35/259 patients were deemed to suffer from secondary-progressive MS with no substantial difference among groups. 24 patients received HE-DMT (mostly fingolimod). Baseline disability burden was rather low in the lights of the long disease duration and the older age (expanded disability status scale (EDSS) score: 3.3/3.4). The study failed to meet its primary endpoint as the hazard ratio for abundance of new disease activity (new MRI lesions and/or clinical relapses) was 2.89 (1.13–7.39) and treatment discontinuation was thus considered inferior to continuation. Of note, the study did not observe relevant differences in disability worsening rates (11.1%/12.3%). The study did not identify differences in terms of adverse events. The DISCOMS extension study provided further insights into the long-term effects of discontinuing DMTs in older, stable MS patients. Over a mean follow-up of 40 months, no relapses were observed, and new MRI lesions remained rare in both groups (1/30 continuers, 2/44 discontinuers). However, time to new disease activity remained significantly shorter in the discontinuation group.

DOT-MS included 189 patients until its premature termination. Among these, 44 patients were assigned to continue treatment whereas 45 patients stopped DMT. Median age was 55/54 years, median disease duration since MS onset was 13.3/14.1 years and the time since last documented clinical relapse was 9.8/9.4 years. The proportion of patients with SPMS was lower than in DISCOMS (4/89 patients). The degree of baseline disability was comparable among groups and resembled baseline parameters from DISCOMS (EDSS: 3.1/3.1). None of the patients received HE-DMT at baseline.

In March 2023, the study was prematurely terminated following an interim analysis that indicated significant disease activity in the discontinuation group. Compared to zero patients in the continuation group, 8/45 stoppers experienced any disease activity (clinical relapses: two patients; significant MRI activity (≥ 3 new lesions or ≥ 2 contrast-enhancing lesions): seven patients). The median follow-up at termination was 15.3 months. Furthermore, 10/45 patients in the discontinuation group had already re-started their DMT. Again, no new safety signals were detected.

In summary, both randomized clinical trials presented here indicated the potential risk of disease reactivation following treatment discontinuation. However, neither study had a sufficient sample size for subgroup analyses, including age strata. The premature termination of DOT-MS further limits the ability to draw definitive conclusions, and caution is required when extrapolating these findings. Given these limitations, direct comparisons between the two studies should be avoided, although some experts have considered the younger age of DOT-MS patients as potential risk factor within the study design.

Whereas these two studies shed light on the disease course following cessation of platform therapies, a knowledge gap remained regarding the long-term disease course of patients stopping HE-DMTs. The (early) use of HE-DMTs is continuously increasing given recent studies indicating their superiority over platform treatments or escalation treatment strategies [7, 8]. We thus decided to summarize results of a recent study from the French MS registry despite its lack of randomization [34] (Table 1). Jouvenot and colleagues identified patients beyond the age of 50 without clinical relapses or new MRI lesions within the past two years while having been on HE-DMT (natalizumab, fingolimod, or rituximab/ocrelizumab) versus those who continued treatment after propensity-score matching (termed OFSEP study within further text). Following propensity-score matching, 154 continuers were compared to 154 stoppers. Unlike DISCOMS and DOT-MS, the authors did not use a non-inferiority testing but included hazard analyses. Mean baseline age was 57.5/57.9 years (HE-DMT discontinuation/HE-DMT continuation), median disease duration was 20.5/19.2 years and time since last clinical relapse and/or MRI activity was 4.4/4.7 years [34]. Of note, baseline disability was higher than in the aforementioned trials (EDSS: 5.0/4.5) and the proportion of patients with SPMS was higher as well (135/308 patients). The mean follow-up was significantly longer in stoppers (3.0 ± 2.2 vs. 1.9 ± 1.9 years). Generally, discontinuation was associated with a significantly higher relapse risk (hazard ratio of 4.1).

Notably, this increased risk was mainly driven by patients stopping natalizumab (HR 7.2 (2.1–24.5)) or fingolimod (HR 4.5 (1.3–15.5)), whereas patients stopping CD20 antibodies did not experience increased relapse rates (HR 1.1 (0.3–4.8)). Also in terms of disability worsening, patients stopping natalizumab (HR: 5.2 (1.5–17.6)) and fingolimod (2.4 (0.9–6.0) appeared more prone compared to patients stopping CD20 therapy (1.9 (0.9–4.3)). However, as this was a registry study the results must be interpreted with caution. Additionally, the limited sample sizes, particularly for specific DMT subgroups, may have reduced statistical power, making it difficult to draw definitive conclusions about the differential relapse and disability risks associated with discontinuing various therapies.

However, this study shows that—in line with DISCOMS and DOT-MS—treatment discontinuation remains associated with the risk of re-emerging disease activity irrespective of age. Of note, the authors observed a time-dependent increase of disease activity following CD20 discontinuation which is in line with the underlying mechanism of action. Similar effects have been observed in patients undergoing extended interval dosing of ocrelizumab before [35]. To address these limitations, future research should prioritize prospective, randomized trials to systematically evaluate HE-DMT discontinuation under controlled conditions. Currently, another randomized clinical trial is conducted evaluating treatment discontinuation in patients with inactive SPMS older than 50 years (STOP-I-SEP; NCT03653273). Primary completion is currently expected in 2026.

Although these studies broadened our understanding of DMT discontinuation, some questions remain unanswered:

First of all, the definition of SPMS remains challenging and despite multiple attempts to establish diagnostic criteria, subjective judgement remains “gold standard” in clinical routine [36].

Recent discoveries however tackled the concept of SPMS as a disease state characterized by disability worsening in absence of focal inflammation. Initially described in RMS patients receiving ocrelizumab [14], disability progression independent of relapse activity (PIRA) was identified as the main driver of disability worsening in all MS phenotypes [13, 37] and was even identified in pediatric-onset MS [38]. Whereas the proportion of patients with SPMS was rather low in DISCOMS and DOT-MS, the OFSEP study included around 40% of patients with SPMS. Unfortunately, none of the studies included any information on whether these patients experienced PIRA within the period in which patients were required to be free of inflammatory activity. Of course, one could argue that the age-related phenomenon of declining inflammatory activity and increasing PIRA which are potentially mediated by immunosenescence and compartimentalization of inflammation within the CNS are common among MS patients [39, 40]. However, at the individual patient level, it remains difficult to make this distinction. One can easily imagine that the relapse risk of a patient whose biological inflammatory activity is present but suppressed by treatment is higher compared to a patient who has genuinely entered the "degenerative disease phase."

Notably, determination of serum biomarkers such as neurofilament light chain serum levels at discontinuation were discussed [41] for improved risk stratification but baseline levels were not predictive of re-emerging disease activity in the few active patients of the DOT-MS trial [42]. This underscores the need to consider additional factors, such as age-related changes and comorbidities, when interpreting biomarker levels. NfL is a well-established marker of inflammatory activity and neuroaxonal damage, but its baseline levels tend to increase with age, potentially limiting its specificity for disease activity [43]. Similarly, GFAP has been proposed as a marker for an increased risk of progressive disease, even in early stages, though it has not yet been clinically approved for routine use [44, 45]. Since the risk of a clinical relapse currently remains inherently linked to treatment discontinuation, it is necessary to understand the interplay of relapses and subsequent disability worsening in (older) MS patients. Whereas the risk for relapse-associated worsening itself was rather low in a recent meta-analysis [37], its abundance increased with age [46] and this is eventually due to reduced brain reserve [47]. To further evaluate the association of relapses and subsequent PIRA, we recently presented data on a prospective multicenter cohort. Here, we found that among patients with a clinical relapse beyond the age of 50, 10.3% of patients suffered from RAW (relapse-associated worsening) and 18.2% subsequently experienced PIRA (compared to 7.7% in a matched cohort without a clinical relapse) [48]. Furthermore, we found that PIRA became even more abundant in an age-dependent manner with a two-fold increase of the odds ratio in patients older than 60 compared to patients aged 50–55. Risk factor analysis also indicated that presence of a DMT at relapse onset was protective against subsequent PIRA. This is in line with previous studies indicating reduced relapse severity and neuroaxonal damage in patients receiving DMT [49, 50]. Finally, we evaluated whether induction of a DMT in a previously untreated patient is sufficient to prevent development of PIRA in the later disease course (as has been performed by 7 patients in DISCOMS study and 10 patients in DOT-MS). Of note, we found that, compared to patients having been on treatment throughout, the risk for PIRA remains substantially elevated and this again points out that active inflammation in the ageing brain precedes subsequent neurodegeneration [51].

Our findings advocate against a “trial and error” approach in terms of drug cessation since re-initiation of a DMT following a clinical relapse in patients might not protect from further disability worsening. Especially in patients receiving a different DMT than the one they stopped or who had longer treatment-free intervals, one has also to consider the “therapeutic lag” before a new DMT exerts its full effect on inflammatory activity and potentially disability worsening [52].

As discussed before, another limitation especially within the OFSEP study is the presence of unknown confounders driving drug cessation and thus the potential introduction of bias. Among patients having stopped treatment in this study, main reasons reported were inactive but progressive disease (22%), adverse events (22%) and “scheduled discontinuation” (35%). As already stated in the introduction, adverse events and safety concerns are likely drivers of drug discontinuation.

Such concerns are potentially fueled by data indicating increased rates of adverse events and comorbidities associated to DMT especially in older patients [53]. Among relevant comorbidities, depressive episodes, as another significant comorbidity, also contributes to the overall complexity of managing MS in older patients [54]. Cardiovascular comorbidities are of particular relevance due to their complex interaction with aging and MS progression, further influencing treatment decisions in this patient population. Furthermore, recent studies shed a light in the complex interplay of cardiovascular diseases and DMT. For example, it was shown that fingolimod substantially increases the risk for CVD in MS patients [55]. Since CVD are more common in older patients, their importance as risk factor for treatment continuation grows with age. Furthermore, polypharmacy often results from CVD as well and this was identified as separate risk factor for drug discontinuation [56, 57]. In our recent study however, we confirmed earlier observations [58] that presence of (multiple) CVD promoted progressive disease following clinical relapses [48]. This was also observed by other groups in 2024 [59]. Thus, the following chapter will review the current evidence on CVD and multiple sclerosis to give a first framework for decision making in (older) patients with CVD and DMT.

## The interplay of CVD, multiple sclerosis and disease-modifying treatment

Generally, the evidence of efficacy and safety profile of DMT in older patients is limited as a result of age restriction in randomized clinical trials (upper age limit usually around 50 to 55 years with few exceptions) [60]. Furthermore, the common age-dependent decline of inflammatory disease activity results in lower effect size in both controlled and observational cohorts and this has repeatedly been interpreted as a “loss of effectiveness” in older patients. The impact of age and immunosenesence/inflammaging on DMT effectiveness however remains to be fully elucidated [61]. Nonetheless, inflammatory disease activity in older patients is associated with unfavourable outcomes and this is also reflected in the worse prognosis of late-onset MS as well [62].

Besides the abovementioned complications of polypharmacy and potential drug interactions, MS and CVD share further associations worth considering. Generally, CVD results in accelerated neurodegeneration and this was impressively underlined by significant elevations of serum neurofilament light chain levels in patients with CVD compared to matched counterparts [63, 64]. These findings are strongly corroborated by increased immunosenescence in patients with CVD [65].

In patients with MS, brain volume loss is further accelerated by CVD indicative of a cumulative effect [66]. In some studies, MS-specific brain atrophy even fell behind age (and potentially vascular)-related brain atrophy [67].

Palladino and colleagues have further demonstrated the impact of CVD in patients with MS impressively as they have shown that MS patients are exceptionally prone to cardiovascular morbidity and mortality compared to non-MS controls in a large national registry [68]. Of note, the higher vascular burden is not unique to MS patients; it mirrors trends observed in other immune-mediated and inflammatory conditions, such as psoriasis, rheumatoid arthritis, and severe atopic eczema, where systemic inflammation is recognized as a significant risk factor for atherosclerosis and cardiovascular disease [69]. Several explanations exist and they include increased levels of blood coagulation factors in patients with MS as well as dysregulation of blood platelets [70, 71]. Additionally, chronic inflammation in MS can lead to endothelial dysfunction, potentially compromising vascular integrity and promoting atherosclerotic changes [72]. Pro-inflammatory cytokines such as tumor necrosis factor-alpha (TNF-α) and interleukin-6 (IL-6) play a central role in this process, as they contribute to increased vascular permeability, oxidative stress, and the activation of coagulation pathways [69, 73]. Lifestyle factors, including reduced physical activity due to MS-related disability, obesity and metabolic alterations may further amplify cardiovascular risk [74, 75]. Taken together, these findings highlight the complex interplay between neuroinflammation, hemostatic disturbances, and cardiovascular risk in MS.

Despite this, MS patients are 40% to 60% less likely to receive appropriate cardiovascular or cerebrovascular treatments than matched controls [76]. This highlights the necessity for vigilance towards CVD in patients with MS and the optimization of risk profiles.

On whether the optimization of CVD directly influences the disease course of MS remains unclear. Several attempts have been made with repurposing drugs like statins for multiple sclerosis, yet all these studies remained inconclusive or negative [77]. In terms of statins, the MS-STAT trial demonstrated that high-dose simvastatin significantly reduced the annualized rate of whole-brain atrophy compared to placebo, while also being well tolerated and safe [78]. However, despite this positive effect on brain atrophy, the trial did not show a significant difference in disability progression or mortality between the simvastatin and placebo groups. Similarly, the MS-STAT2 trial, a multicentre, randomized controlled, double-blind study, included 964 patients with non-active secondary progressive multiple sclerosis who were treated with either high-dose simvastatin (80 mg per day) or placebo for 54 months [79]. The primary outcome of confirmed disability progression at 48 months did not show a significant difference between the simvastatin and placebo groups. Similarly, mortality rates were not significantly different between the two groups, which is surprising given the neuroprotective and cerebrovascular benefits expected from statins.

What however has not been evaluated systematically to date is whether DMT can reduce the increased risk for CVD in patients with MS back to levels of matched general population patients. Several studies however indicated that treatment with various immunomodulatory substances alleviated the cardiovascular risk profile [80] and randomized controlled trials evaluating the anti-interleukin-1β antibody canakinumab improved cardiovascular disease outcomes [81]. Unfortunately, treatment did not reduce mortality. Vice versa, cancer patients treated with immune-checkpoint inhibitors, which per definition accelerate systemic inflammation, show increased risks for cardiovascular events [82]. Randomized clinical trials in MS have not yielded specific information on the protection from CVD by DMT, yet within these trials, many efforts were undertaken to avoid presence of CVD by inclusion of younger and healthy patients. Additionally, follow-up periods were likely too short before patients entered open-label extension studies. Thus, future research including observational data from larger registries appears warranted.

To sum up, CVD indicate unfavourable disease courses in patients with MS and the optimization of risk profiles is recommendable. Although it currently remains unclear whether DMT themselves influence CVD positively, they should not be stopped solely for reduction of polypharmacy in order to allow a more “holistic” approach to brain protection. However, potential side effects must also be considered, making an individual risk/benefit assessment essential.

## Conclusions

**Fig. 1:**
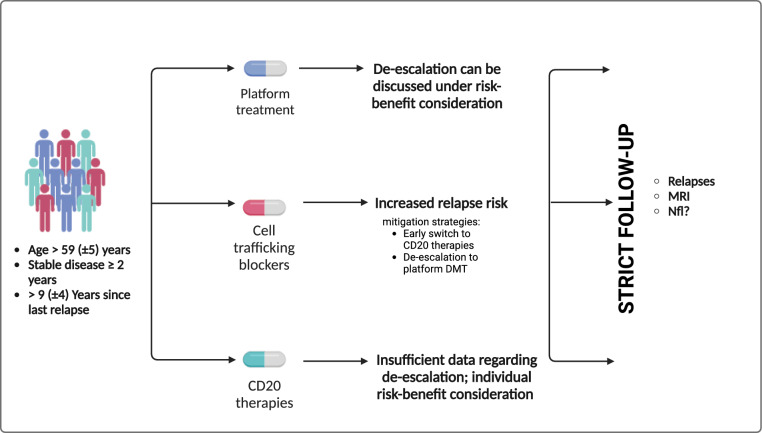
Framework for patient selection criteria and discontinuation decisions

Discontinuation of DMT in patients with MS has been discussed for many years although motivations to stop treatments have changed. Apart from special situations, as for example washout of cell-trafficking inhibitors, evidence regarding the safety of a treatment stop remains unclear. Evidence from the DISCOMS, DOT-MS, and OFSEP studies suggests that older patients with stable disease for more than two years and no recent relapses—typically around a mean age of 59 years—may be suitable candidates for discontinuation (Fig. 1). For patients receiving platform therapies, discontinuing treatment can be discussed with the patient based on a risk–benefit assessment. In contrast, those on cell-trafficking inhibitors face a higher risk of relapse. In such cases, strategies like early transition to CD20 therapy or de-escalation to platform disease-modifying therapies (DMTs) may help mitigate this risk.

For CD20 treatment, current data is insufficient to support de-escalation. Thus, de-escalation can only be considered in discussion with the patient, taking the risk–benefit profile into account. Regardless of the treatment type, close monitoring after treatment discontinuation remains essential to detect potential disease reactivation and enable timely intervention if needed.

Although the majority of patients in selected collectives (older age, stable disease for many years) did not experience disease re-activation during the follow-up period of respective studies, the impact of re-emerging disease can be serious on single patients. The avoidance of polypharmacy in older patients with MS and treatment-requiring comorbidities was identified as important driver of treatment discontinuation. However, since comorbidities, especially CVDs, negatively affect disease courses in patients with MS, treatment discontinuation in patients should be well-weighted.

## Data Availability

Not applicable.

## Abbreviations

DMT: Disease-modifying therapy
EDSS: Expanded disability status scale
HE-DMT: High-efficacy disease-modifying therapy
MS: Multiple sclerosis
PwMS: Patients with multiple sclerosis
PIRA: Progression independent of relapse activity
RAW: Relapse-associated worsening
